# Characterizing fetoplacental response to acute maternal hyperoxygenation in suspected coarctation of aorta using fetal cardiac MRI

**DOI:** 10.1002/uog.29303

**Published:** 2025-07-19

**Authors:** M. P. M. van Poppel, J. K. Steinweg, T. Woodgate, V. Zidere, T. Day, J. Hutter, R. Razavi, J. M. Simpson, T. Vigneswaran, K. Pushparajah, D. F. A. Lloyd

**Affiliations:** ^1^ Research Department of Early Life Imaging School of Biomedical Engineering and Imaging Sciences, King's College London London UK; ^2^ Department of Congenital Heart Disease Evelina London Children's Hospital London UK

**Keywords:** aortic coarctation, fetal development, fetal imaging, heart defect, congenital, magnetic resonance imaging, placental circulation, prenatal diagnosis

## Abstract

**Objective:**

To characterize the fetoplacental response to acute maternal hyperoxygenation (AMH) in fetuses with suspected coarctation of the aorta (CoA) using fetal cardiac magnetic resonance imaging (MRI).

**Methods:**

Pregnant women carrying a fetus with suspected CoA were recruited prospectively from a tertiary fetal cardiology center as part of the Fetal Imaging with Maternal Oxygen (FIMOx) study between February 2019 and April 2022. MRI included phase‐contrast flow measurements and oxygen‐sensitive T2* sequences to assess placental and fetal brain oxygenation. Results were analyzed on a groupwise basis, comparing true‐positive CoA cases (requiring surgical CoA repair ≤ 28 days after birth) to false positives.

**Results:**

Fetal MRI with AMH was performed in 20 pregnancies (mean ± SD gestational age, 33.0 ± 1.3 weeks), of which five had CoA and 15 were false positives. In both groups, AMH significantly increased the placental T2* signal but had no effect on cerebral T2*. Increased pulmonary blood flow with AMH was associated with reduced right–left shunting at the foramen ovale (*r* = −0.74, *P* < 0.001), but not with increased ascending aortic flow (*r* = 0.36, *P* = 0.177). Compared with baseline, AMH increased anterograde flow at the aortic isthmus from −2 (interquartile range (IQR), −57 to 10) mL/kg/min to 32 (IQR, −14 to 48) mL/kg/min (*P* < 0.001); however, this was associated with a reduction in superior vena cava flow (median, −29% (IQR, −41% to 0%); *P* < 0.001) and not with changes in ascending aortic flow. No differences in baseline or AMH phase‐contrast or T2* values usefully distinguished true‐ and false‐positive cases in this pilot study.

**Conclusions:**

AMH leads to a significant reduction in cerebral blood flow in fetuses with suspected CoA, which is the primary driver of changes in flow at the aortic isthmus. Future studies should consider the effects of maternal oxygen on the entirety of fetoplacental circulation, particularly for cases in which chronic maternal hyperoxygenation administration is being considered. © 2025 The Author(s). *Ultrasound in Obstetrics & Gynecology* published by John Wiley & Sons Ltd on behalf of International Society of Ultrasound in Obstetrics and Gynecology.

## INTRODUCTION

The concept of delivering maternal face‐mask oxygen during pregnancy was first explored in the 1990s to treat intrauterine growth restriction^1^. Recently, new applications for maternal hyperoxygenation (MH) have been proposed in the assessment of fetal congenital heart disease (CHD)^2^, ^3^, ^4^, ^5^, fetal brain development^6^, ^7^ and placental function^8^, ^9^, ^10^.

Acute maternal hyperoxygenation (AMH) is known to reduce fetal pulmonary vascular resistance in late‐gestation fetuses^11^, thereby increasing pulmonary blood flow (PBF), left ventricular (LV) preload, and flow through the LV and aorta^12^, ^13^, ^14^. Echocardiographic assessment of flow at the aortic isthmus (*Q*_Ist_) has been posited as a means of non‐invasively evaluating the LV response to AMH in the context of impaired LV filling from an aneurysmal atrial septum (AAS)^5^, ^15^. Fetuses with coarctation of the aorta (CoA), a notoriously challenging condition to predict before birth^16^, demonstrate abnormal LV myocardial deformation on echocardiography and fetal magnetic resonance imaging (MRI)^17^, ^18^. However, no studies to date have investigated the AMH response in discriminating between true‐positive (TP) and false‐positive (FP) CoA cases in fetal life.

In our previous work, we showed that both TP and FP CoA fetuses demonstrate a similar pattern of flow redistribution that differs significantly from a healthy control population^16^. More recently, using three‐dimensional statistical shape analysis, we showed an intrinsic relationship between flow and function at the aortic/ductal junction^19^, in keeping with theories on the etiology of CoA^20^. Separately, oxygen‐sensitive T2* sequences have been used to estimate tissue oxygenation in the fetal brain and placenta in fetuses with CHD, both at baseline^21^ and with the application of AMH^22^. Finally, chronic MH, whereby maternal oxygen is supplied for several hours a day for a period of weeks or months, has been proposed as a means of stimulating growth of the fetal left heart^23^, ^24^. However, concerns have been raised regarding the effects of chronic MH on the fetal cerebral circulation, with potential impacts on brain development^25^, ^26^, ^27^.

In this pilot study, we sought to characterize the comprehensive fetoplacental response to AMH in a cohort of fetuses with suspected CoA to determine whether the AMH response could: (1) help to understand the pathophysiology present in both TP and FP CoA fetuses; and (2) identify relevant biomarkers for future studies that could help to discriminate between these two groups.

## METHODS

### Study design

Pregnant women carrying a fetus with suspected CoA were recruited prospectively from a large tertiary fetal cardiology center (Evelina London Children's Hospital/Guy's and St Thomas' NHS Foundation Trust, London, UK) from February 2019 to April 2022. All patients provided written informed consent before entering the study (Fetal Imaging with Maternal Oxygen (FIMOx)) and ethical approval was granted by the London–South East Research Ethics Committee (REC:17/LO/0282). Cases were identified and offered fetal cardiac MRI at the discretion of the attending fetal cardiologist. Maternal exclusion criteria included weight > 150 kg, abdominal girth > 130 cm, severe claustrophobia, inability to give informed consent and age < 18 years at the time of referral. In view of the requirement for supplemental maternal oxygen, additional exclusion criteria for this study included severe maternal respiratory pathology and/or use of specific chemotherapeutic agents. Suspected CoA pregnancies were classified as TP or FP, where the former was defined as neonatal CoA requiring surgical repair ≤ 28 days after birth, once postnatal data were available. All infants were followed up for at least 12 months to observe for late‐developing CoA^28^.

### MRI acquisition

All MRI data were acquired using a Philips Ingenia 1.5‐Tesla machine (Philips, Best, The Netherlands). Participants were imaged in the left lateral position. Phase contrast MRI sequences were acquired as follows: repetition time (TR), 6.78 ms; echo time (TE), 3.15 ms; matrix size, 240 × 240; field of view (FOV), 240 mm; slice thickness, 3 mm; simulated R–R interval, 545 ms. Total scan time was limited to 60 min. Retrospective gating was performed using metric optimized gating, according to methodology published previously^29^. Flows were measured at baseline (in room air) and following administration of > 15 min of 10 L/min oxygen inhaled via non‐rebreather face mask in six main fetal vessels: ascending aorta (*Q*_AAo_), superior vena cava (*Q*_SVC_), descending aorta (*Q*_DAo_), main pulmonary artery (*Q*_MPA_), arterial duct (*Q*_AD_) and umbilical vein (*Q*_UV_). *Q*_SVC_ was used as a surrogate for cerebral blood flow, as described previously^30^. *Q*_Ist_, PBF, foramen ovale flow (*Q*_FO_) and combined ventricular output (CVO) were derived from the measured flows according to methodology published previously, with 3% CVO used to represent estimated coronary flow^16^. T2* data were obtained using a free‐breathing multi‐echo gradient‐echo (MEGE) echo planar imaging pulse sequence with four echo times between 7 ms and 160 ms covering the entire uterus and the brain in coronal orientation to the mother. Scan resolution was 1.5 × 1.5 × 2.5 mm, typical FOV was 256 × 256 × 40 mm, TEs were 7.3, 57.8, 108.3 and 154.6 ms, and TR was 10–12 s.

### MRI data processing

Flow measurements were performed using the CVI42 platform (Circle Cardiovascular Imaging Inc., Calgary, Canada). All flows were indexed to the estimated fetal weight, calculated from a single stack of two‐dimensional MRI images with whole‐uterus coverage via an established volume–weight conversion formula^31^. Mean T2* values were extracted from T2* maps generated using an in‐house monoexponential fitting script in MATLAB (MathWorks Inc., Natick, MA, USA) as published previously^32^, based on automatic segmentation (with manual corrections where needed) for the placenta and fetal brain^33^. The mean T2* value averaged across the first five dynamics (at baseline) and last five dynamics (after > 15 min of AMH) was used for subsequent analysis.

### Statistical analysis

Phase‐contrast MRI flow analysis was performed using IBM SPSS Statistics for Mac (version 28.0.1.1 (14); IBM Corp., Chicago, IL, USA). Descriptive data for continuous variables are presented as mean ± SD or median (interquartile range (IQR)). Categorical data are presented as *n* (%). Baseline characteristics were compared between groups using independent or paired‐sample *t*‐tests or the Mann–Whitney *U‐*test for continuous variables; Fisher's exact test or Pearson's chi‐square test was used for categorical variables. *P*< 0.05 was considered statistically significant. Correlations were assessed using Spearman's correlation coefficient.

## RESULTS

Twenty‐five patients were recruited for fetal cardiac MRI with AMH. Five cases were excluded because only baseline phase‐contrast MRI data were available due to time constraints and/or excessive fetal motion in the latter part of the study. The mean ± SD gestational age at fetal cardiac MRI in the remaining 20 patients was 33.0 ± 1.3 weeks. All included cases were singleton pregnancies. Neonatal CoA (surgical repair of CoA ≤ 28 days after birth) was confirmed in five (25%) patients and the remaining 15 (75%) cases were classified as FP. Patient characteristics, including comorbidities, are shown in Table 1. T2* sequences were available in 13 patients (eight FP, five TP). Nine (60%) cases in the FP cohort were reported to have an AAS on at least one previous fetal echocardiogram, compared to none in the TP group (*P* = 0.020). One FP patient was found postnatally to have a small anomalous pulmonary vein (left upper pulmonary vein to innominate vein), which was not hemodynamically significant. No other discrepancies were noted between prenatal and postnatal diagnosis of CoA.

**Table 1:** Characteristics of pregnancies with true‐ or false‐positive diagnosis of fetal coarctation of aorta (CoA)

Characteristic	False‐positive (*n* = 15)	True‐positive (*n* = 5)	*P*
GA at fetal cardiac MRI (weeks)	33.2 ± 1.1	32.3 ± 1.7	0.185
EFW on MRI (kg)	2.17 ± 0.39	2.14 ± 0.21	0.874
GA at birth (weeks)	39.1 ± 1.2	39.1 ± 0.4	0.937
Birth weight (kg)	3.47 ± 0.45	3.02 ± 1.20	0.053
Days at surgery	—	4 (3–6)	—
Late CoA*	0 (0)	—	—
Aneurysmal atrial septum	9 (60)	0 (0)	0.020
Bicuspid aortic valve	1 (7)	2 (40)	0.071
Mitral valve abnormality	0 (0)	1 (20)	0.076
Ventricular septal defect	2 (13)	2 (40)	0.388
Bilateral superior vena cava	1 (7)	0 (0)	0.554
Growth restriction†	1 (7)	0 (0)	0.554
Genetic abnormality	0 (0)	1 (20)	0.076

Data are given as mean ± SD, median (interquartile range) or *n* (%).

* > 28 days to 12 months.

† Birth weight < 5^th^ centile. EFW, estimated fetal weight; GA, gestational age; MRI, magnetic resonance imaging.

### General observations

**Figure 1:**
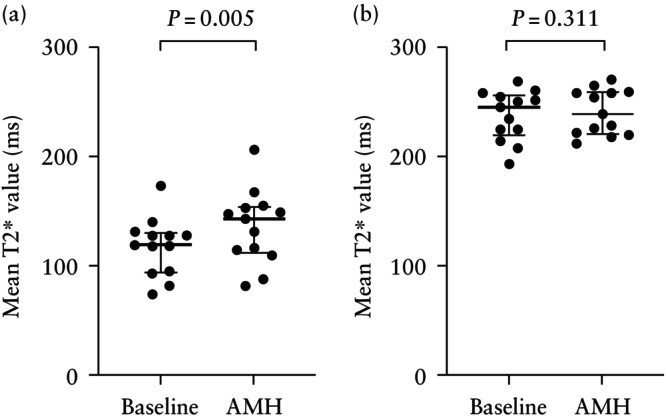
Change in mean T2* signal (reflecting proportion of saturated hemoglobin) averaged over five consecutive dynamics in the placenta (a) and brain (b) at baseline and after acute maternal hyperoxygenation (AMH) in the entire cohort of fetuses with suspected coarctation of aorta. Horizontal bars and error bars represent median and interquartile range, respectively.

**Figure 2:**
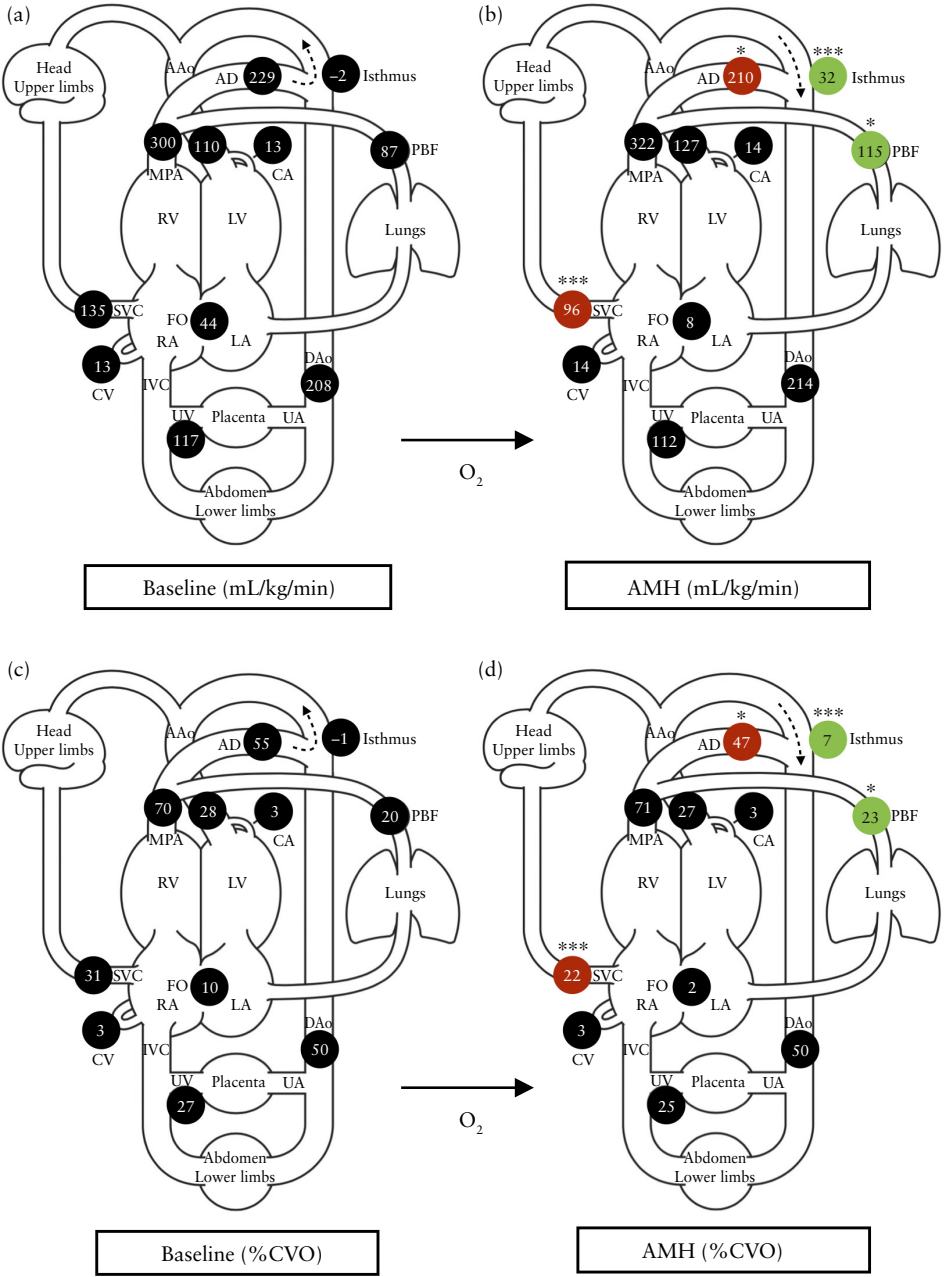
Schematic diagrams showing flow measurements derived from phase‐contrast magnetic resonance imaging, indexed to estimated fetal weight (a,b) and presented as percentage of combined ventricular output (CVO) (c,d) at baseline (a,c) and after acute maternal hyperoxygenation (AMH) (b,d) for entire cohort of fetuses with suspected coarctation of aorta. Significant increases are shown in green and significant decreases in red (*P < 0.05, ***P < 0.001). Dashed arrows represent the direction of blood flow at the aortic isthmus. AAo, ascending aorta; AD, arterial duct; CA, coronary artery; CV, coronary vein; DAo, descending aorta; FO, foramen ovale; IVC, inferior vena cava; LA, left atrium; LV, left ventricle; MPA, main pulmonary artery; O_2_, oxygen; PBF, pulmonary blood flow; RA, right atrium; RV, right ventricle; SVC, superior vena cava; UA, umbilical artery; UV, umbilical vein.

**Figure 3:**
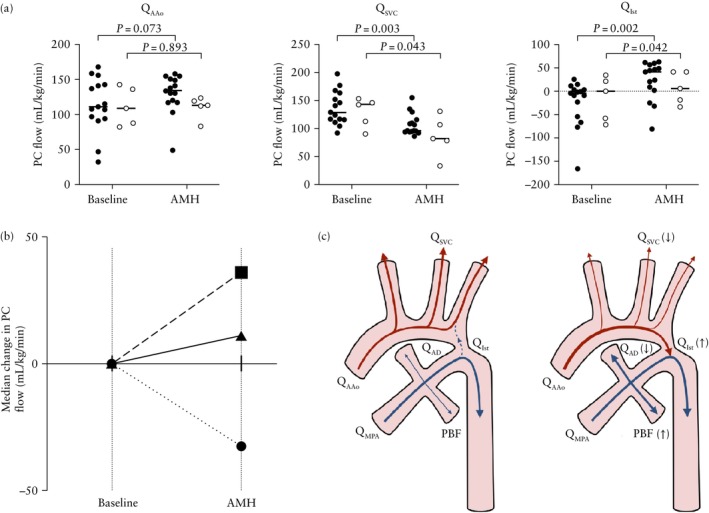
(a) Scatter plots showing median phase‐contrast (PC) magnetic resonance imaging flow in ascending aorta (*Q*_AAo_), superior vena cava (*Q*_SVC_) and aortic isthmus (*Q*_Ist_) at baseline and after acute maternal hyperoxygenation (AMH) in cases with false‐positive (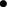) or true‐positive (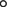) diagnosis of coarctation of aorta. (b) Median change in *Q*_AAo_ (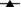), *Q*_SVC_ (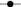) and *Q*_Ist_ (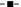) following administration of AMH for the entire cohort. (c) Flow before (left panel) and after (right panel) AMH for the entire cohort. Red and blue arrows represent the distribution of blood flow from the aorta and main pulmonary artery, respectively, and line thickness corresponds to magnitude of flow. Significant increases and decreases are indicated by up and down arrows, respectively. PBF, pulmonary blood flow; *Q*_AD_, arterial duct flow; *Q*_MPA_, main pulmonary artery flow.

Following administration of AMH, there was a significant increase in placental T2* value across the cohort (*P* = 0.005); however, fetal brain T2* was unchanged (*P* = 0.331) (Table 2 and Figure 1). Median values for phase‐contrast flow before and after AMH are shown in Table 2, and Figures 2 and 3. Across the cohort as a whole, compared with baseline, administration of AMH was associated with increased PBF (median, 87 (IQR, 36–111) mL/kg/min *vs* 115 (IQR, 70–164) mL/kg/min; *P* = 0.048), reduced *Q*_AD_ (median, 229 (IQR, 188–281) mL/kg/min *vs* 210 (IQR, 161–256) mL/kg/min; *P* = 0.010) and reduced cerebral blood flow (*Q*_SVC_) (median, 135 (IQR, 114–153) mL/kg/min *vs* 96 (IQR, 92–114) mL/kg/min; *P* < 0.001), the latter representing a median reduction of −29% (IQR, −41% to 0%; *P* < 0.001) from baseline. Increased PBF following AMH correlated with reduced right–left shunting at the foramen ovale (*r* = −0.74, *P* < 0.001), but not with changes in *Q*_AAo_ (*r* = 0.36, *P* = 0.177). Administration of AMH was also associated with a significant increase in *Q*_Ist_ from a median of −2 (IQR, −57 to 10) mL/kg/min to 32 (IQR, −14 to 48) mL/kg/min (*P* < 0.001). This correlated with reduced *Q*_SVC_ (*r* = −0.55, *P* = 0.011), but not with changes in *Q*_AAo_ (*r* = 0.301, *P* = 0.211).

**Table 2:** Measurements derived from magnetic resonance imaging at baseline and after acute maternal hyperoxygenation (AMH) in fetuses with suspected coarctation of aorta

	False‐positive				True‐positive				All cases			
Variable	*n*	Baseline	AMH	*P*	*n*	Baseline	AMH	*P*	*n*	Baseline	AMH	*P*
T2* (ms)												
Placenta	8	107 (85 to 128)	116 (94 to 149)	0.050	5	128 (119 to 152)	153 (137 to 181)	0.043	13	120 (94 to 130)	143 (112 to 154)	0.005
Brain	8	251 (222 to 258)	257 (223 to 260)	0.161	5	225 (209 to 252)	226 (221 to 249)	0.893	13	246 (220 to 257)	239 (221 to 259)	0.311
Phase‐contrast flow (mL/kg/min)												
Ascending aorta	15	111 (94 to 143)	134 (117 to 151)	0.073	5	109 (85 to 140)	113 (98 to 122)	0.893	20	110 (92 to 142)	127 (114 to 147)	0.121
Main pulmonary artery	15	298 (269 to 343)	332 (296 to 369)	0.140	5	306 (264 to 409)	284 (276 to 342)	0.465	20	300 (270 to 354)	322 (285 to 358)	0.409
Superior vena cava	15	129 (116 to 164)	96 (94 to 116)	0.003	5	143 (102 to 150)	82 (56 to 119)	0.043	20	135 (114 to 153)	96 (92 to 114)	< 0.001
Descending aorta	14	207 (188 to 236)	221 (197 to 282)	0.311	5	215 (181 to 235)	205 (177 to 226)	0.465	19	208 (184 to 234)	214 (196 to 270)	0.523
Arterial duct	14	236 (184 to 294)	220 (160 to 259)	0.048	5	215 (189 to 267)	205 (151 to 234)	0.043	19	229 (188 to 281)	210 (161 to 256)	0.010
Aortic isthmus	15	−4 (−54 to 4)	42 (2 to 57)	0.002	5	0 (−65 to 29)	6 (−27 to 42)	0.042	20	−2 (−57 to 10)	32 (−14 to 48)	< 0.001
Pulmonary blood flow	13	82 (50 to 102)	117 (64 to 178)	0.046	5	92 (25 to 195)	99 (75 to 149)	0.684	18	87 (36 to 111)	115 (70 to 164)	0.048
Foramen ovale	14	40 (−19 to 66)	12 (−63 to 60)	0.221	5	44 (−76 to 60)	−13 (−42 to 24)	0.500	19	44 (−55 to 64)	8 (−42 to 56)	0.184
Umbilical vein	13	97 (88 to 121)	111 (85 to 133)	0.649	5	135 (113 to 168)	115 (102 to 169)	0.500	18	117 (92 to 128)	112 (96 to 132)	1.000
CVO	15	410 (371 to 468)	487 (420 to 513)	0.033	5	405 (384 to 551)	426 (410 to 471)	0.686	20	408 (377 to 493)	478 (413 to 508)	0.113

Data given as median (interquartile range). CVO, combined ventricular output.

### True‐ and false‐positive CoA

Indexed *Q*_UV_ was lower in the FP group compared with the TP group at baseline (median, 97 (IQR, 88–121) mL/kg/min *vs* 135 (IQR, 113–168) mL/kg/min; *P* = 0.026). There were no other significant differences between groups before the administration of AMH. Both groups independently showed the same pattern of response to AMH in terms of placental and brain T2* values and flow changes in *Q*_SVC_ and *Q*_Ist_; however, while the increase in PBF was significant for the FP cohort, this was not the case in the TP cohort (Table 2). There was also a significant increase in CVO in FP fetuses, from a median of 410 (IQR, 371–468) mL/kg/min at baseline to 487 (IQR, 420–513) mL/kg/min after AMH (*P* = 0.033), which was not present in the TP group. In both groups, increasing PBF correlated with reduced (right–left) *Q*_FO_ (FP group: *r* = −0.70, *P* = 0.004; TP group: *r* = −0.98, *P* = 0.005) but not to changes in *Q*_AAo_ (FP group: *r* = −0.12, *P* = 0.661; TP group: *r* = −0.68, *P* = 0.216).

### Aneurysmal atrial septum

Subdividing the FP group into those with aneurysmal septum (FP/AAS; *n* = 9) and those without (FP/noAAS; *n* = 6) showed that baseline *Q*_UV_ was significantly lower in the FP/AAS group compared with that in the FP/noAAS group (median, 92 (IQR, 83–114) mL/kg/min *vs* 135 (IQR, 113–268) mL/kg/min; *P* = 0.030). There were no other differences between these two subgroups prior to the administration of AMH. In the FP/AAS group, median (right–left) *Q*_FO_ reduced significantly with AMH, from 53 (IQR, 20–67) mL/kg/min to 12 (IQR, −92 to 63) mL/kg/min (*P* = 0.038). No significant difference in *Q*_FO_ was noted in the FP/noAAS group. There was a non‐significant trend towards increasing *Q*_AAo_ in the FP/AAS group, from a median of 107 (IQR, 69–139) mL/kg/min at baseline to 130 (IQR, 110–150) mL/kg/min after AMH (*P* = 0.069). Median *Q*_AAo_ in the FP/noAAS group was 129 (IQR, 108–161) mL/kg/min at baseline and 136 (IQR, 119–156) mL/kg/min after AMH (*P* = 0.458).

## DISCUSSION

In this exploratory study, we described the effects of AMH on the fetoplacental circulation in a cohort of fetuses with suspected CoA. To our knowledge, this is the first study to combine MRI assessment of the fetal circulatory response to AMH with oxygen‐sensitive T2* sequences of the placenta and fetal brain.

Given that maternal hemoglobin is nearly fully saturated at baseline (in contrast to fetal hemoglobin) the increased placental T2* signal with AMH can be attributed to increased oxygen saturation on the chorionic or ‘fetal‐side’ blood pool, providing useful *in vivo* evidence of effective oxygen transfer to the fetus as a mechanism for downstream hemodynamic responses. Across the cohort as a whole, AMH was associated with increased PBF and reduced *Q*_AD_. While increased PBF correlated with reduced right–left shunting at the atrial level, we observed no significant correlation with increased *Q*_AAo_. Broadly, both FP and TP CoA fetuses showed the same overall pattern; however, the increase in PBF was not statistically significant in the TP group when analyzed on a groupwise basis. Future studies with higher numbers of participants are needed to establish whether this a consistent pattern in CoA fetuses.

Perhaps the most significant observation in this study is that the increase in anterograde flow at the aortic isthmus following the administration of AMH was driven primarily by a reduction in cerebral blood flow, with no relationship to *Q*_AAo_. This was a highly consistent pattern across the cohort, which, in combination with the relatively stable T2* signal in the fetal brain, suggests an important cerebral autoregulatory response to modulate cerebral oxygen delivery during AMH. This is in keeping with previous animal studies that showed reduced cerebral blood flow in fetal lambs when exposed to hyperoxia^26^, and with studies in human pregnancies that demonstrated increased cerebrovascular resistance (measured by middle cerebral artery pulsatility on ultrasound) following administration of AMH^34^. However, whilst the importance of the aortic isthmus in this context is well recognized^34^, ^35^, the interdependence of aortic, cerebral and isthmal flow is a novel observation in the context of AMH, and an important consideration when assessing downstream fetal responses. For example, in fetuses with a small or borderline left ventricle associated with AAS, measurement of blood flow patterns at the isthmus has been proposed as a means to assess LV filling when challenged with AMH^5^, ^15^. Our results suggest that *Q*_Ist_ may be an unreliable marker of LV output in isolation given the simultaneous effects on isthmal blood flow caused by autoregulation of cerebral oxygen delivery with AMH.

Whilst our study only assessed the acute (≤ 60 min) fetoplacental response to AMH, there are potential implications for the use of chronic intermittent MH, posited as a means of encouraging growth of left heart structures (including the aortic isthmus) via the mechanism of increasing PBF, LV preload and flow through the left heart^23^, ^24^. As well as the effects of reduced cerebral blood flow on wider fetal hemodynamics, there is also a theoretical reduction in delivery of other critical substrates, such as glucose, with the potential to impact fetal brain growth over longer periods^25^, ^26^. A recent phase 1 clinical trial of chronic MH in fetuses with single ventricle CHD showed a non‐significant trend towards reduced *Q*_SVC_, but no differences in early neurological and neurodevelopmental outcomes^36^. However, the same study also showed little or no sustained effect of MH on the fetal circulation, raising important questions for future research in this area.

In keeping with previous echocardiographic research^15^, we noted a greater increase in *Q*_AAo_ in FP cases with AAS, although this did not reach significance in the current study. However, we did observe that *Q*_UV_ was significantly lower in the AAS group at baseline. Given that flow across the foramen ovale in fetal life is predominantly placental in origin (via the ductus venosus), this potentially suggests a more complex relationship between *Q*_UV_ and atrial septal development, and represents an interesting area for future exploration.

Finally, our study is not the first to observe different T2* responses in the placenta and brain to AMH. You *et al*.^22^ showed a similar increase in placental T2* across a range of CHD subtypes following the administration of AMH, but with a relatively static cerebral T2* signal, as per the current study. Interestingly, the authors observed an increase in cerebral T2* in fetuses with single ventricle physiology and aortic obstruction, suggesting that the AMH response may be influenced by other anatomical and hemodynamic factors. In our own work, we have previously shown a significant reduction in baseline T2* signal in the placenta across a range of fetal CHD subtypes, particularly in the FP CoA group^33^. Indeed it should be noted that some of the circulatory features observed in this study, such as retrograde aortic arch flow, are known to be associated independently with underlying placental insufficiency^1^, which can be subclinical^37^. Future studies looking in greater detail at specific subgroups of CHD may help to understand these differences and their implications for the use of MH.

### Future directions

The observed reduction in cerebral blood flow, coupled with the static T2* signal in the fetal brain, implies increased oxygen content in the ascending aorta following administration of AMH. Novel MRI methods, such as T1/T2 mapping, may provide the means to directly measure intravascular oxygen saturations in the future^38^, alongside emerging methods to assess changes in the fetal circulation, such as ventricular volumetry^39^, functional data^18^ and four‐dimensional flow^40^. Additionally, the distribution patterns observed in cases with suspected CoA share similar characteristics to the so‐called ‘brain sparing’ response in fetuses with known placental insufficiency^1^, ^16^, ^19^, ^37^, and a range of clinical and subclinical placental abnormalities have been described in fetuses with CHD, including CoA^33^, ^41^. A holistic imaging protocol, using advanced MRI with ultrasonography to assess the entirety of the fetal and placental circulation, may offer further diagnostic insights.

### Limitations

In previous work, we have shown that FP fetuses have significantly reduced flow through the left heart at baseline compared with healthy control fetuses^2^; therefore, the inclusion of a control group may add further insights into both FP and TP CoA cases. This is an area of active investigation in our group. Moreover, while fetal cardiac MRI methods have shown rapid development over the last decade, many of these techniques are not yet widely available. Future studies examining the response to AMH using both MRI and ultrasound could enhance the applicability of these findings in a clinical setting.

### Conclusions

AMH leads to a reduction in cerebral blood flow in fetuses with suspected CoA, which contributes to significantly increased forward flow at the aortic isthmus on fetal cardiac MRI. Future studies should consider the effects of maternal oxygen on the entirety of fetoplacental circulation, particularly for cases in which chronic MH administration is being considered.

## Data Availability

The data that support the findings of this study are available on request from the corresponding author. The data are not publicly available due to privacy or ethical restrictions.
